# Crystal structure of {2-[({2-[(2-amino­eth­yl)amino]­eth­yl}imino)­meth­yl]pheno­lato}aqua­copper(II) bromide

**DOI:** 10.1107/S1600536814017590

**Published:** 2014-08-16

**Authors:** Nataliya I. Plyuta, Julia A. Rusanova, Svitlana R. Petrusenko, Irina V. Omelchenko

**Affiliations:** a Taras Shevchenko National University of Kyiv, Department of Inorganic Chemistry, Volodymyrska str. 64/13, 01601 Kyiv, Ukraine; b Institute for Scintillation Materials, "Institute for Single Crystals", National Academy of Sciences of Ukraine, Lenina ave. 60, Kharkov 61001, Ukraine

**Keywords:** crystal structure, copper(II) complex, Schiff base ligand, bromide, hydrogen bonding

## Abstract

In the mononuclear copper(II) title complex, [Cu(C_11_H_16_N_3_O)(H_2_O)]Br, the Cu^II^ atom is coordinated by one O and three N atoms of the Schiff base ligand that forms together with one water mol­ecule a slightly distorted [CuN_3_O_2_] square-pyramidal polyhedron. The deviation of the Cu^II^ atom from the mean equatorial plane is 0.182 (2) Å. The equatorial plane is nearly coplanar to the aromatic ring of the ligand [angle between planes = 10.4 (1)°], and the water molecule is situated in the apical site. All coordinating atoms (except the imine nitro­gen) and the bromide ion contribute to the formation of the N—H⋯Br, O—H⋯Br and O—H⋯O hydrogen bonds, which link mol­ecules into chains along [01-1].

## Related literature   

For structures isotypic with that of the title compound, see: Zhu *et al.* (2002 ▶, 2004 ▶); He (2003 ▶). For the direct synthesis of copper-containing coordination compounds using the salt route, see: Kovbasyuk *et al.* (1997 ▶); Pryma *et al.* (2003 ▶); Buvaylo *et al.* (2005 ▶); Nikitina *et al.* (2008 ▶); Vassilyeva *et al.* (1997 ▶); Makhankova *et al.* (2002 ▶). For the direct synthesis of polynuclear copper-containing complexes, see: Nesterova (Pryma) *et al.* (2004 ▶); Nesterova *et al.* (2005 ▶).
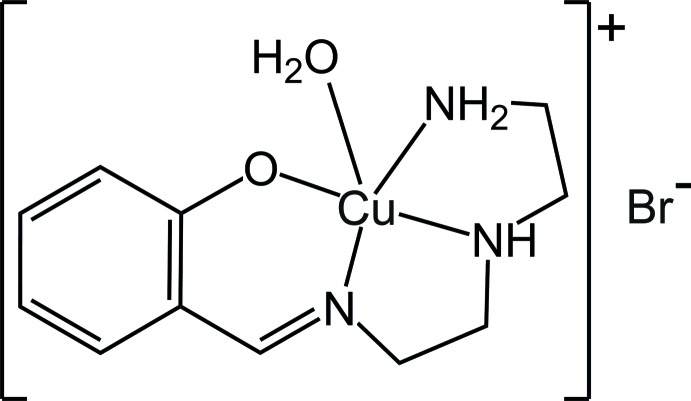


## Experimental   

### Crystal data  

[Cu(C_11_H_16_N_3_O)(H_2_O)]Br
*M* *_r_* = 367.73
Monoclinic, 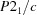

*a* = 9.2226 (11) Å
*b* = 14.0333 (13) Å
*c* = 10.9206 (11) Å
β = 102.355 (11)°
*V* = 1380.7 (3) Å^3^
*Z* = 4
Mo *K*α radiation
μ = 4.47 mm^−1^
*T* = 293 K
0.40 × 0.40 × 0.40 mm

### Data collection  

Agilent Xcalibur Sapphire3 diffractometer
Absorption correction: multi-scan (*CrysAlis PRO*; Agilent, 2011 ▶) *T*_min_ = 0.268, *T*_max_ = 0.268
7804 measured reflections
4004 independent reflections
2334 reflections with *I* > 2σ(*I*)
*R*_int_ = 0.045

### Refinement  

*R*[*F* ^2^ > 2σ(*F* ^2^)] = 0.052
*wR*(*F* ^2^) = 0.097
*S* = 0.95
4004 reflections
163 parameters
H-atom parameters constrained
Δρ_max_ = 0.98 e Å^−3^
Δρ_min_ = −0.38 e Å^−3^

### 

Data collection: *CrysAlis PRO* (Agilent, 2011 ▶); cell refinement: *CrysAlis PRO*; data reduction: *CrysAlis RED* (Agilent, 2011 ▶); program(s) used to solve structure: *SHELXTL* (Sheldrick, 2008 ▶); program(s) used to refine structure: *OLEX2* (Dolomanov *et al.*, 2009 ▶); molecular graphics: *SHELXTL*; software used to prepare material for publication: *publCIF* (Westrip, 2010 ▶).

## Supplementary Material

Crystal structure: contains datablock(s) I, New_Global_Publ_Block. DOI: 10.1107/S1600536814017590/rn2126sup1.cif

Structure factors: contains datablock(s) I. DOI: 10.1107/S1600536814017590/rn2126Isup2.hkl

**Fig. 1.**
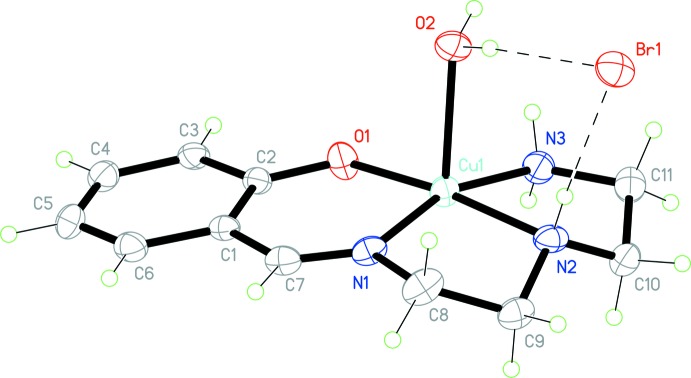
Structure of the title compound, with displacement ellipsoids drawn at the 50% probability level for non-H atoms with hydrogen bonds shown as dashed lines.

. DOI: 10.1107/S1600536814017590/rn2126fig1.tif
Structure of the title compound, with displacement ellipsoids drawn at the 50% probability level for non-H atoms with hydrogen bonds shown as dashed lines.

**Fig. 2.**
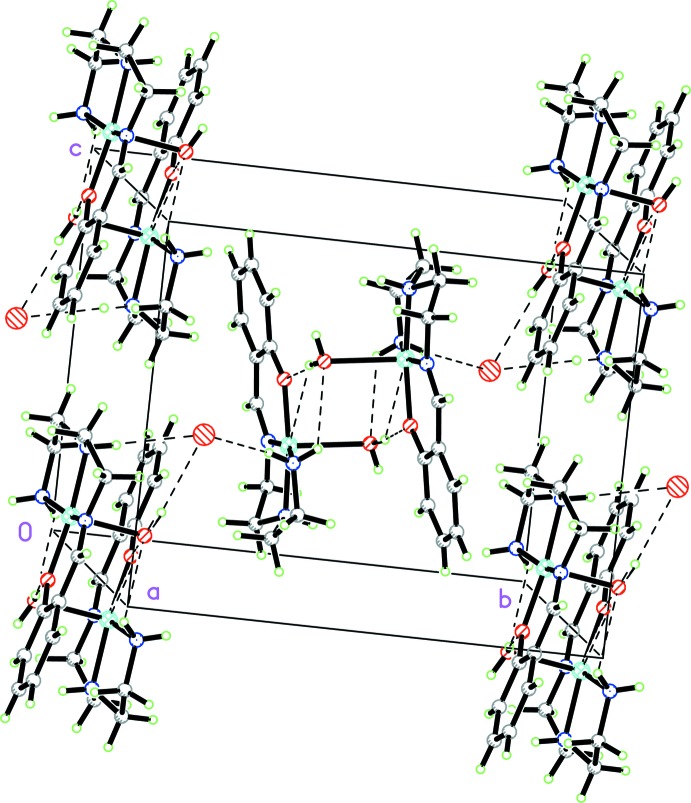
Crystal packing of the title compound with hydrogen bonds shown as dashed lines.

. DOI: 10.1107/S1600536814017590/rn2126fig2.tif
Crystal packing of the title compound with hydrogen bonds shown as dashed lines.

CCDC reference: 1017209

Additional supporting information:  crystallographic information; 3D view; checkCIF report

## Figures and Tables

**Table 1:** Hydrogen-bond geometry (Å, °)

*D*—H⋯*A*	*D*—H	H⋯*A*	*D*⋯*A*	*D*—H⋯*A*
O2—H2*OB*⋯Br1	0.82	2.51	3.323 (3)	173
N2—H2*N*⋯Br1	0.85	2.58	3.429 (3)	177
O2—H2*OA*⋯O1^i^	0.82	1.90	2.712 (4)	171
N3—H3*NA*⋯Br1^ii^	0.85	2.68	3.499 (3)	164

Symmetry codes: (i) 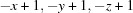; (ii) 
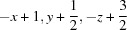
.

## supplementary crystallographic information

### S1. Comment

It has been shown that the direct synthesis is an efficient method to obtain novel homo and heterometallic mono/polynuclear coordination compounds (Kovbasyuk *et al.*, 1997; Vassilyeva *et al.*, 1997; Makhankova *et al.*, 2002; Pryma *et al.*, 2003; Nesterova (Pryma) *et al.*, 2004; Nesterova *et al.*, 2005; Buvaylo *et al.*, 2005; Nikitina *et al.*, 2008). The title compound, [Cu(C_11_H_18_N_3_O_2_)(H_2_O)]Br, was obtained unintentionally as the product of an attempted synthesis of a Cu/ Mn heterometallic complex using zerovalent copper and manganese powders, ammonium bromide, salicylic aldehyde and diethylenetriamine in dimethylformamide on air.

As shown in Fig. 1, the Cu^II^ atom has a slightly distorted square-pyramidal geometry formed by one oxygen and three nitrogen atoms of the Schiff base ligand as well one oxygen atom of the coordinated water molecule. The deviation of the copper atom from the mean equatorial plane is 0.182 (2) Å. The range of Cu–N and Cu–O bond distances in the equatorial plane is 1.918 (3) - 2.018 (3) Å, while the Cu–O axial distance is 2.333 (2) Å. These data are in a good agreement with literature values (Zhu *et al.*,2002, 2004; He *et al.*, 2003). The equatorial plane is nearly coplanar to the aromatic ring of the ligand [angle between planes is 10.4 (1)°].

In the crystal, OH···O hydrogen bonds form molecular dimers. OH···Br and NH···Br hydrogen bonds link the dimers into chains along the [011] crystallographic direction (See Table containing Hydrogen-bond geometry and Fig.2).

### S2. Experimental

The title compound was synthesized by addition of manganese powder 0.055 g (1 mmol), copper powder 0.06 g (1 mmol) and NH_4_Br 0.392 g (4 mmol) to the previously prepared Schiff base ligand solution [mixture of salicylic aldehyde 0.21 ml (2 mmol) and diethylenetriamine 0.108 ml (1 mmol) in dimethylformamide (10 ml) which was stirred about 15 min at 323–333 K until the mixture turned yellow]. The total reaction mixture was stirred magnetically for 4 h until the complete dissolution of manganese and copper powders was observed. Dark green crystals that precipitated after 1 day were collected by filtration and dried in air.

### S3. Refinement

Structure was solved by direct method and refined against F^2^ with anisotropic refinement for all non-hydrogen atoms. All H atoms were placed in idealized positions (C–H = 0.93 – 0.97 Å, O–H = 0.82 Å, N–H 0.85 Å) and constrained to ride on their parent atoms, with *U*_iso_ = 1.2Ueq (except *U*_iso_ = 1.5Ueq for water).

### Crystal data

**Table:**

[Cu(C_11_H_16_N_3_O)(H_2_O)]Br	*F*(000) = 740
*M**_r_* = 367.73	*D*_x_ = 1.769 Mg m^−^^3^
Monoclinic, *P*2_1_/*c*	Mo *K*α radiation, λ = 0.71073 Å
Hall symbol: -P 2ybc	Cell parameters from 1502 reflections
*a* = 9.2226 (11) Å	θ = 2.9–32.2°
*b* = 14.0333 (13) Å	µ = 4.47 mm^−^^1^
*c* = 10.9206 (11) Å	*T* = 293 K
β = 102.355 (11)°	Block, green
*V* = 1380.7 (3) Å^3^	0.40 × 0.40 × 0.40 mm
*Z* = 4	

### Data collection

**Table:**

Agilent Xcalibur Sapphire3 diffractometer	4004 independent reflections
Radiation source: Enhance (Mo) X-ray Source	2334 reflections with *I* > 2σ(*I*)
Graphite monochromator	*R*_int_ = 0.045
Detector resolution: 16.1827 pixels mm^-1^	θ_max_ = 30.0°, θ_min_ = 2.9°
ω scans	*h* = −7→12
Absorption correction: multi-scan (*CrysAlis PRO*; Agilent, 2011)	*k* = −16→19
*T*_min_ = 0.268, *T*_max_ = 0.268	*l* = −15→12
7804 measured reflections	

### Refinement

**Table:**

Refinement on *F*^2^	Primary atom site location: structure-invariant direct methods
Least-squares matrix: full	Secondary atom site location: difference Fourier map
*R*[*F*^2^ > 2σ(*F*^2^)] = 0.052	Hydrogen site location: inferred from neighbouring sites
*wR*(*F*^2^) = 0.097	H-atom parameters constrained
*S* = 0.95	*w* = 1/[σ^2^(*F*_o_^2^) + (0.0308*P*)^2^] where *P* = (*F*_o_^2^ + 2*F*_c_^2^)/3
4004 reflections	(Δ/σ)_max_ = 0.001
163 parameters	Δρ_max_ = 0.98 e Å^−^^3^
0 restraints	Δρ_min_ = −0.38 e Å^−^^3^

### Special details

**Table:**

Experimental. Absorption correction: CrysAlis PRO (Agilent, 2011) Empirical absorption correction using spherical harmonics, implemented in SCALE3 ABSPACK scaling algorithm.
Geometry. All e.s.d.'s (except the e.s.d. in the dihedral angle between two l.s. planes) are estimated using the full covariance matrix. The cell e.s.d.'s are taken into account individually in the estimation of e.s.d.'s in distances, angles and torsion angles; correlations between e.s.d.'s in cell parameters are only used when they are defined by crystal symmetry. An approximate (isotropic) treatment of cell e.s.d.'s is used for estimating e.s.d.'s involving l.s. planes.
Refinement. Refinement of *F*^2^ against ALL reflections. The weighted *R*-factor *wR* and goodness of fit *S* are based on *F*^2^, conventional *R*-factors *R* are based on *F*, with *F* set to zero for negative *F*^2^. The threshold expression of *F*^2^ > σ(*F*^2^) is used only for calculating *R*-factors(gt) *etc*. and is not relevant to the choice of reflections for refinement. *R*-factors based on *F*^2^ are statistically about twice as large as those based on *F*, and *R*- factors based on ALL data will be even larger.

### Fractional atomic coordinates and isotropic or equivalent isotropic displacement parameters (Å^2^)

**Table:**

	*x*	*y*	*z*	*U*_iso_*/*U*_eq_	
Cu1	0.67287 (6)	0.58118 (3)	0.65849 (4)	0.03317 (14)	
Br1	0.70894 (6)	0.32849 (3)	0.91251 (4)	0.04828 (15)	
O1	0.6489 (3)	0.61004 (19)	0.4836 (2)	0.0412 (7)	
O2	0.6414 (3)	0.41770 (17)	0.6240 (2)	0.0401 (7)	
H2OA	0.5548	0.4032	0.5949	0.060*	
H2OB	0.6614	0.3912	0.6920	0.060*	
N1	0.8883 (4)	0.5872 (2)	0.6891 (3)	0.0346 (8)	
N2	0.7031 (4)	0.5675 (2)	0.8462 (3)	0.0387 (8)	
H2N	0.7059	0.5079	0.8604	0.046*	
N3	0.4595 (4)	0.6068 (2)	0.6646 (3)	0.0399 (8)	
H3NA	0.4358	0.6632	0.6411	0.048*	
H3NB	0.4087	0.5679	0.6132	0.048*	
C1	0.9071 (4)	0.6273 (2)	0.4771 (3)	0.0302 (8)	
C2	0.7532 (5)	0.6287 (2)	0.4219 (3)	0.0313 (9)	
C3	0.7118 (5)	0.6529 (2)	0.2942 (3)	0.0349 (9)	
H3	0.6117	0.6542	0.2556	0.042*	
C4	0.8153 (5)	0.6747 (3)	0.2252 (4)	0.0424 (11)	
H4	0.7837	0.6912	0.1412	0.051*	
C5	0.9668 (5)	0.6727 (3)	0.2784 (4)	0.0463 (11)	
H5	1.0368	0.6864	0.2309	0.056*	
C6	1.0092 (5)	0.6498 (3)	0.4026 (4)	0.0419 (10)	
H6	1.1100	0.6491	0.4394	0.050*	
C7	0.9648 (5)	0.6070 (2)	0.6085 (4)	0.0366 (9)	
H7	1.0673	0.6085	0.6364	0.044*	
C8	0.9587 (5)	0.5675 (3)	0.8202 (4)	0.0467 (11)	
H8A	1.0528	0.6007	0.8429	0.056*	
H8B	0.9771	0.4997	0.8320	0.056*	
C9	0.8548 (5)	0.6011 (3)	0.9012 (4)	0.0411 (10)	
H9A	0.8868	0.5762	0.9855	0.049*	
H9B	0.8561	0.6702	0.9058	0.049*	
C10	0.5790 (5)	0.6137 (3)	0.8863 (4)	0.0440 (11)	
H10A	0.5935	0.6822	0.8899	0.053*	
H10B	0.5728	0.5915	0.9691	0.053*	
C11	0.4385 (5)	0.5896 (3)	0.7935 (4)	0.0492 (11)	
H11A	0.4132	0.5233	0.8027	0.059*	
H11B	0.3576	0.6287	0.8092	0.059*	

### Atomic displacement parameters (Å^2^)

**Table:**

	*U*^11^	*U*^22^	*U*^33^	*U*^12^	*U*^13^	*U*^23^
Cu1	0.0316 (3)	0.0396 (3)	0.0258 (2)	0.0004 (2)	0.00050 (19)	0.0017 (2)
Br1	0.0532 (3)	0.0425 (2)	0.0456 (3)	−0.0024 (2)	0.0028 (2)	0.0084 (2)
O1	0.0259 (16)	0.0651 (18)	0.0285 (14)	−0.0052 (14)	−0.0029 (12)	0.0099 (13)
O2	0.0341 (17)	0.0430 (15)	0.0378 (15)	−0.0046 (13)	−0.0039 (12)	−0.0007 (13)
N1	0.032 (2)	0.0361 (17)	0.0306 (17)	0.0052 (15)	−0.0046 (14)	−0.0041 (15)
N2	0.053 (2)	0.0278 (16)	0.0327 (18)	−0.0054 (16)	0.0025 (16)	0.0013 (14)
N3	0.037 (2)	0.0425 (18)	0.0396 (19)	−0.0004 (16)	0.0076 (16)	−0.0011 (15)
C1	0.027 (2)	0.0261 (17)	0.036 (2)	0.0005 (17)	0.0039 (17)	−0.0055 (16)
C2	0.033 (2)	0.0300 (19)	0.031 (2)	−0.0043 (18)	0.0052 (17)	−0.0009 (16)
C3	0.035 (2)	0.037 (2)	0.029 (2)	−0.0076 (18)	0.0001 (17)	−0.0034 (16)
C4	0.058 (3)	0.036 (2)	0.035 (2)	−0.004 (2)	0.014 (2)	−0.0020 (18)
C5	0.047 (3)	0.047 (2)	0.052 (3)	0.002 (2)	0.026 (2)	−0.012 (2)
C6	0.032 (3)	0.040 (2)	0.055 (3)	−0.0030 (19)	0.012 (2)	−0.010 (2)
C7	0.024 (2)	0.036 (2)	0.046 (2)	0.0033 (17)	−0.0014 (19)	−0.0055 (18)
C8	0.047 (3)	0.052 (3)	0.032 (2)	0.014 (2)	−0.0111 (19)	0.0004 (19)
C9	0.047 (3)	0.043 (2)	0.028 (2)	0.003 (2)	−0.0043 (19)	0.0004 (18)
C10	0.054 (3)	0.045 (2)	0.036 (2)	−0.002 (2)	0.015 (2)	−0.0047 (18)
C11	0.048 (3)	0.059 (3)	0.043 (3)	−0.008 (2)	0.016 (2)	−0.005 (2)

### Geometric parameters (Å, º)

**Table:**

Cu1—O1	1.919 (3)	C2—C3	1.407 (5)
Cu1—N1	1.945 (3)	C3—C4	1.371 (5)
Cu1—N3	2.016 (3)	C3—H3	0.9300
Cu1—N2	2.018 (3)	C4—C5	1.395 (6)
Cu1—O2	2.333 (2)	C4—H4	0.9300
O1—C2	1.313 (4)	C5—C6	1.367 (6)
O2—H2OA	0.8197	C5—H5	0.9300
O2—H2OB	0.8159	C6—H6	0.9300
N1—C7	1.271 (5)	C7—H7	0.9300
N1—C8	1.466 (5)	C8—C9	1.512 (6)
N2—C10	1.461 (5)	C8—H8A	0.9700
N2—C9	1.477 (5)	C8—H8B	0.9700
N2—H2N	0.8495	C9—H9A	0.9700
N3—C11	1.481 (5)	C9—H9B	0.9700
N3—H3NA	0.8455	C10—C11	1.503 (6)
N3—H3NB	0.8494	C10—H10A	0.9700
C1—C6	1.407 (5)	C10—H10B	0.9700
C1—C2	1.418 (5)	C11—H11A	0.9700
C1—C7	1.447 (5)	C11—H11B	0.9700
			
O1—Cu1—N1	93.31 (12)	C2—C3—H3	119.1
O1—Cu1—N3	95.17 (12)	C3—C4—C5	121.3 (4)
N1—Cu1—N3	162.80 (13)	C3—C4—H4	119.3
O1—Cu1—N2	173.18 (12)	C5—C4—H4	119.3
N1—Cu1—N2	85.17 (14)	C6—C5—C4	117.8 (4)
N3—Cu1—N2	84.65 (14)	C6—C5—H5	121.1
O1—Cu1—O2	93.61 (10)	C4—C5—H5	121.1
N1—Cu1—O2	99.09 (11)	C5—C6—C1	122.8 (4)
N3—Cu1—O2	95.29 (11)	C5—C6—H6	118.6
N2—Cu1—O2	93.20 (10)	C1—C6—H6	118.6
C2—O1—Cu1	127.7 (2)	N1—C7—C1	126.1 (4)
Cu1—O2—H2OA	112.6	N1—C7—H7	117.0
Cu1—O2—H2OB	107.8	C1—C7—H7	117.0
H2OA—O2—H2OB	104.6	N1—C8—C9	108.0 (3)
C7—N1—C8	121.5 (4)	N1—C8—H8A	110.1
C7—N1—Cu1	126.0 (3)	C9—C8—H8A	110.1
C8—N1—Cu1	112.5 (3)	N1—C8—H8B	110.1
C10—N2—C9	118.1 (3)	C9—C8—H8B	110.1
C10—N2—Cu1	108.4 (2)	H8A—C8—H8B	108.4
C9—N2—Cu1	107.2 (2)	N2—C9—C8	109.0 (3)
C10—N2—H2N	112.2	N2—C9—H9A	109.9
C9—N2—H2N	104.5	C8—C9—H9A	109.9
Cu1—N2—H2N	105.7	N2—C9—H9B	109.9
C11—N3—Cu1	109.3 (3)	C8—C9—H9B	109.9
C11—N3—H3NA	111.3	H9A—C9—H9B	108.3
Cu1—N3—H3NA	110.3	N2—C10—C11	108.4 (3)
C11—N3—H3NB	111.0	N2—C10—H10A	110.0
Cu1—N3—H3NB	105.5	C11—C10—H10A	110.0
H3NA—N3—H3NB	109.3	N2—C10—H10B	110.0
C6—C1—C2	119.0 (4)	C11—C10—H10B	110.0
C6—C1—C7	117.9 (4)	H10A—C10—H10B	108.4
C2—C1—C7	123.1 (4)	N3—C11—C10	109.5 (4)
O1—C2—C3	118.9 (4)	N3—C11—H11A	109.8
O1—C2—C1	123.7 (3)	C10—C11—H11A	109.8
C3—C2—C1	117.3 (4)	N3—C11—H11B	109.8
C4—C3—C2	121.7 (4)	C10—C11—H11B	109.8
C4—C3—H3	119.1	H11A—C11—H11B	108.2

### Hydrogen-bond geometry (Å, º)

**Table:**

*D*—H···*A*	*D*—H	H···*A*	*D*···*A*	*D*—H···*A*
O2—H2*OB*···Br1	0.82	2.51	3.323 (3)	173
N2—H2*N*···Br1	0.85	2.58	3.429 (3)	177
O2—H2*OA*···O1^i^	0.82	1.90	2.712 (4)	171
N3—H3*NA*···Br1^ii^	0.85	2.68	3.499 (3)	164

Symmetry codes: (i) −*x*+1, −*y*+1, −*z*+1; (ii) −*x*+1, *y*+1/2, −*z*+3/2.
