# Supplementary figures and images for: Crystal structure of {2-[({2-[(2-amino­eth­yl)amino]­eth­yl}imino)­meth­yl]pheno­lato}aqua­copper(II) bromide
Source: Acta Crystallogr Sect E Struct Rep Online. 2014 Aug 16;70(Pt 9):m330–1. doi: 10.1107/S1600536814017590 (PMC4186199; doi:10.1107/S1600536814017590)

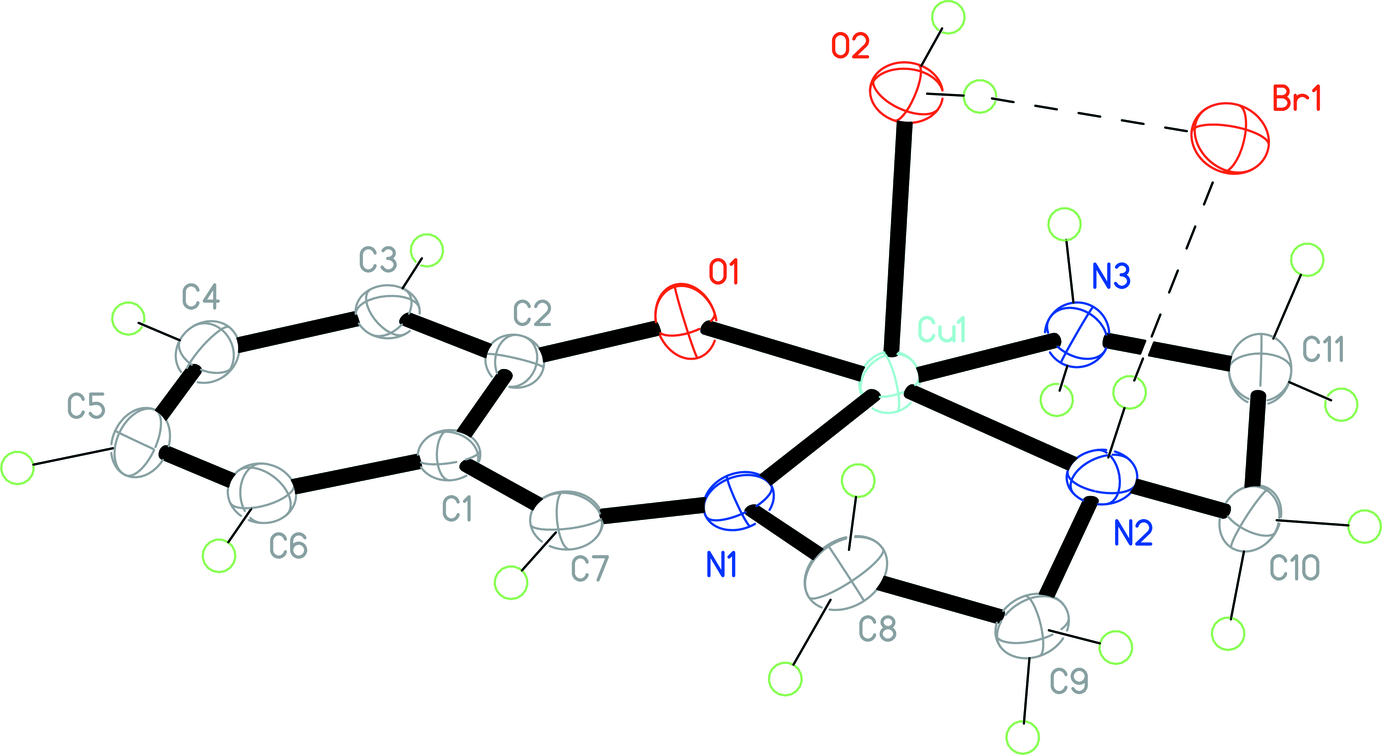

Supplement: Supplementary file 3 [file e-70-0m330-fig1.tif]

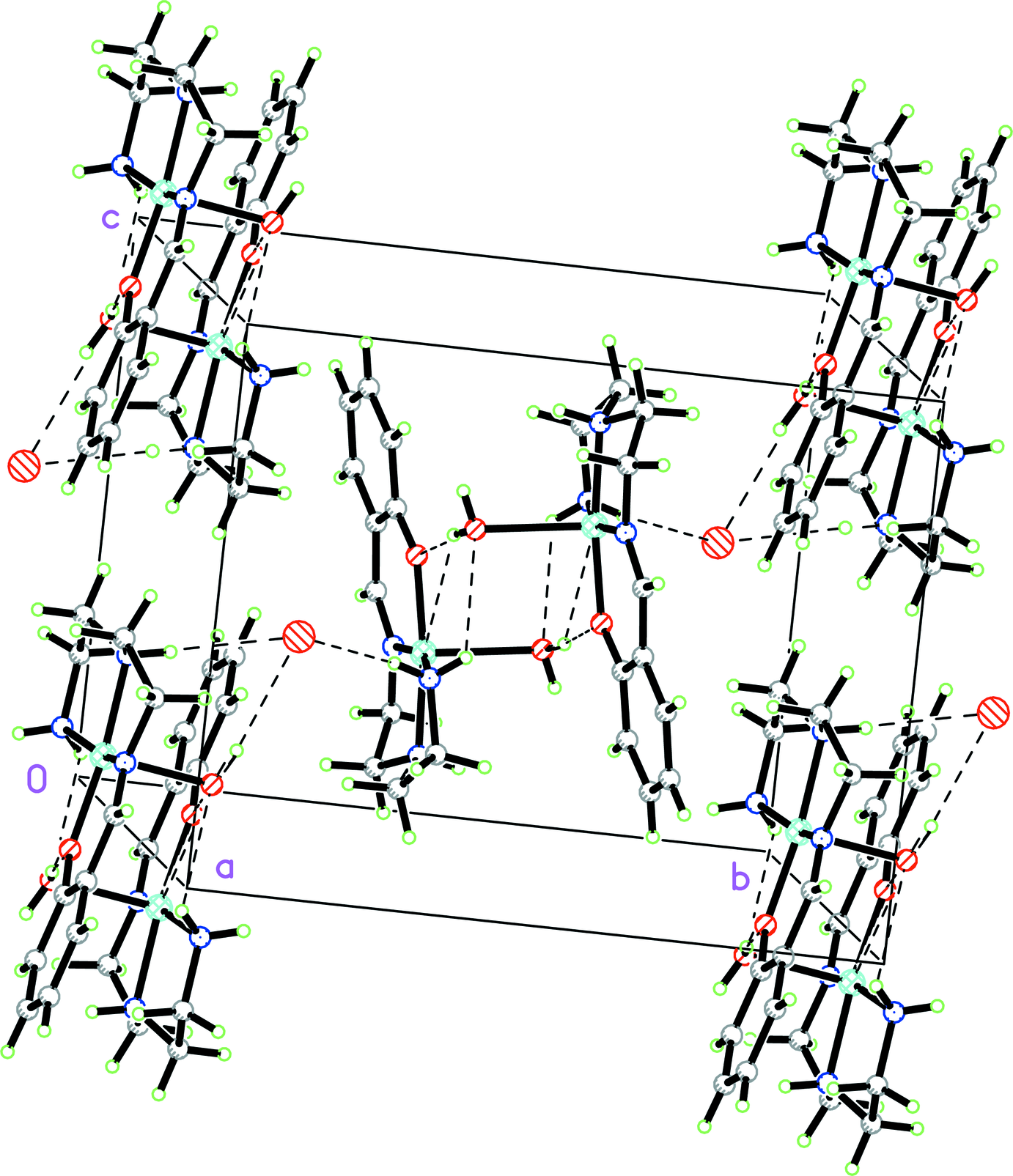

Supplement: Supplementary file 4 [file e-70-0m330-fig2.tif]
